# Magnetic reversal in perpendicularly magnetized antidot arrays with intrinsic and extrinsic defects

**DOI:** 10.1038/s41598-019-49869-5

**Published:** 2019-09-16

**Authors:** Michal Krupinski, Pawel Sobieszczyk, Piotr Zieliński, Marta Marszałek

**Affiliations:** Institute of Nuclear Physics Polish Academy of Sciences, Radzikowskiego 152, 31–342 Kraków, Poland

**Keywords:** Magnetic properties and materials, Magnetic properties and materials, Ferromagnetism, Surfaces, interfaces and thin films

## Abstract

Defects can significantly affect performance of nanopatterned magnetic devices, therefore their influence on the material properties has to be understood well before the material is used in technological applications. However, this is experimentally challenging due to the inability of the control of defect characteristics in a reproducible manner. Here, we construct a micromagnetic model, which accounts for intrinsic and extrinsic defects associated with the polycrystalline nature of the material and with corrugated edges of nanostructures. The predictions of the model are corroborated by the measurements obtained for highly ordered arrays of circular Co/Pd antidots with perpendicular magnetic anisotropy. We found that magnetic properties, magnetic reversal and the evolution of the domain pattern are strongly determined by density of defects, heterogeneity of nanostructures, and edge corrugations. In particular, an increase in the Néel domain walls, as compared to Bloch walls, was observed with a increase of the antidot diameters, suggesting that a neck between two antidots can behave like a nanowire with a width determined by the array period and antidot size. Furthermore, the presence of edge corrugations can lead to the formation of a network of magnetic bubbles, which are unstable in non-patterned flat films.

## Introduction

Periodic arrays of ferromagnetic nanostructures have been intensively investigated over the recent years for their fundamental and applied properties. Their importance for fundamental research stems from the low dimensionality and a large contribution of surface and edge regions resulting in magnetic properties that are not observed in bulk or continuous flat films. A particularly interesting example is an array of antidots, which consists of ordered holes embedded in a continuous magnetic film. The presence of the nonmagnetic holes induces novel magnetic domain configurations and domain wall pinning, which affect static and dynamic properties of the system^1^. In particular, the magnetization reversal, the coercive field, and the effective magnetic anisotropy depend on the array geometry^2–6^, the period^7^, the antidot size^3,8^ and shape^9,10^, as well as the order parameter^8^.

The possibility of tuning of the magnetic properties makes the antidot arrays a potential candidate for the microwave devices^11^, magnetic sensors^12^, directed transport of magnetic nanoparticles^13^, and high-density storage media^14,15^. In the latter case, systems with perpendicular magnetic anisotropy (PMA) are especially interesting, since they offer both a high stability and a possibility of rapid magnetic reversal^16^. However, their practical use is possible only after a careful adjustment of their properties. The optimization of the composition, morphology, and the geometrical parameters is required, but the device performance could also be affected by the defects and inhomogeneity, which can be unavoidable in the large scale materials. The defects may have a strong impact on the magnetic properties^17^, and thus their influence should be understood well before the nanopatterned material is used in technological applications.

The defects can be divided into two groups: intrinsic, which are distributed randomly in the pristine material as a result of e.g. chemical contamination or layer roughness, and extrinsic, which are non-material-dependent, are introduced by patterning, and appear on the edges of nanostructures^17^. Many multilayer systems with perpendicular magnetic anisotropy, such as Co/Pd, Co/Pt, and Co/Ni, are particularly sensitive to extrinsic defects and even only a small modification of their thickness or deterioration of interface quality can lead to the loss of perpendicular magnetization^18^. As a result, the magnetic anisotropy of the edge material can change significantly, resulting in a change of the reversal process for the entire antidot array^17,19^.

The difficulty in studying of the influence of edge defects on the magnetic reversal behavior lies in the inability to regulate their characteristics experimentally in a reproducible manner. The width of the defected edge, its roughness and composition are determined by the chosen patterning method and cannot be easily varied. Additionally, the experimentally measured parameters such as coercivity or effective anisotropy are a superposition of contributions from different defects, which often cannot be studied separately^20^.

A significant progress in exploring this issue may be achieved by employing computational tools. For this purpose, we built a micromagnetic model of antidot array with perpendicular magnetization that takes into account all major intrinsic and extrinsic defects associated with the polycrystalline character of the material, including defected edges with modified magnetic properties. Additionally, the heterogeneity of the patterning process giving rise to the variably-sized antidots was also studied. We validated our model experimentally on Co/Pd multilayers patterned by nanosphere lithography, a representative system with perpendicular magnetic anisotropy. So far, little data exist on the influence of the defects and patterning heterogeneities on the properties of antidot arrays with PMA, and there is no comprehensive micromagnetic model of such systems. One of the goals of this work is to provide such a model and demonstrate the effect of defects on the magnetic reversal of the multilayers with PMA.

## Results and Discussion

### Reference flat multilayers

**Figure 1:**
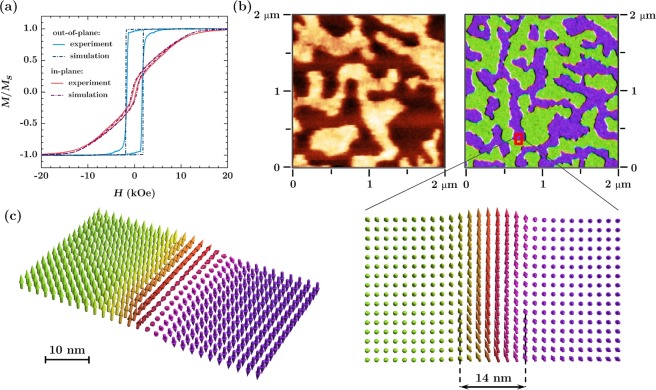
(**a**) Simulated and measured at 5 K M-H loops for a reference flat Co/Pd multilayer with the magnetic fields applied in-plane and out-of-plane; (**b**) comparison of domain patterns measured with MFM and simulated for the same reference multilayer; (**c**) magnetic moments configuration for a region chosen from the magnetic domain map (b, right panel) showing the Bloch domain wall with the thickness of 14 nm.

The M-H loops for a flat reference sample measured by SQIUD magnetometry at 5 K are shown in Fig. 1a. They are compared to hysteresis loops simulated with the material parameters specified in the Experimental and Calculations section. Coercivity field, loop squareness, and effective anisotropy constants for the experiment and the simulation are given in Table 1. One can notice a reasonable agreement between the shape of the loops as well as the calculated and measured values of the magnetic parameters. This similarity suggests an accurate selection of the intrinsic parameters of the material and indicates that the structure of the adopted model is realistic.

**Table 1:** Comparison of measured and simulated coercive field *H*_*C*_, squareness and effective anisotropy constant *K*_*eff*_ for a flat Co/Pd multilayer.

	experiment		simulation	
	out-of-plane	in-plane	out-of-plane	in-plane
*H*_*C*_	1700 Oe	40 Oe	1850 Oe	50 Oe
squareness	0.97	0.11	0.99	0.14
*K*_*eff*_	0.37 MJ/m^3^		0.37 MJ/m^3^	

The in-plane hysteresis loop exhibits two distinct magnetization phases: one that quickly reverses at fields close to the coercive field, and another that linearly approaches the saturation. The first phase originates from “easy-switchers”, grains with zero uniaxial anisotropy, which easily change the direction of magnetization and orient it according to the shape anisotropy of the thin film. The reproduction of this phase in M-H loops is impossible without incorporating into the model the defected grains with low *K*_*U*_. The second phase is related to grains with a strong perpendicular anisotropy, where magnetization progressively rotates under the application of an in-plane magnetic field^21^.

The magnetization reversal curve for the out-of-plane magnetic field is dominated by domain nucleation, propagation, and annihilation. The grains with reduced anisotropy or with a significantly misoriented direction of the easy-axis are the source of domain nucleation, and by an exchange coupling with the surrounding material they start a fast magnetic reversal of the whole sample. Such process was analyzed in detail by Shaw *et al*.^22^, who showed that it strongly affects the value of the out-of-plane coercive field. This suggests that in order to obtain a better match between the measured and simulated coercivity one should focus on a more precise determination of the contributions of the defected grains and the dispersion of their magnetization easy axes.

The domain pattern for a flat reference sample as measured by MFM is shown in Fig. 1b. The sample was imaged after out-of-plane ac demagnetization, which resulted in irregular domains typical for the systems with PMA. The scan is compared with the simulation result showing a similar configuration of the domains. The small discrepancy in the sizes and domain wall length may be a result of the differences in demagnetization process. Due to a long calculation time the simulations had to be limited to several relaxation cycles, whereas in reality the sample was subjected to several dozen cycles of field switching. Another explanation could stem from the differences in temperature, since the imaging was performed at 300 K. The model does not take into account thermal fluctuations, while it is known that they may have a substantial impact on the magnetic domain configuration and size^23–26^.

Example of the magnetic moment configuration for a selected region of the sample is presented in Fig. 1c. The transition region between two domains is shown, where the magnetization rotates in the domain wall plane, characteristic for Bloch type walls, as expected for the thin films with PMA. The mean domain wall thickness determined from the simulations is about 14 nm, in agreement with estimation given by the formula^27^:

1 $$\delta \approx \pi \sqrt{\frac{A}{{K}_{u}}}.$$

where *A* is the exchange stiffness coefficient and *K*_*U*_ the uniaxial anisotropy constant, which gives the value of *δ* ≈ 10 nm for *A* and *K*_*U*_ values mentioned previously.

### Arrays of antidots

**Figure 2:**
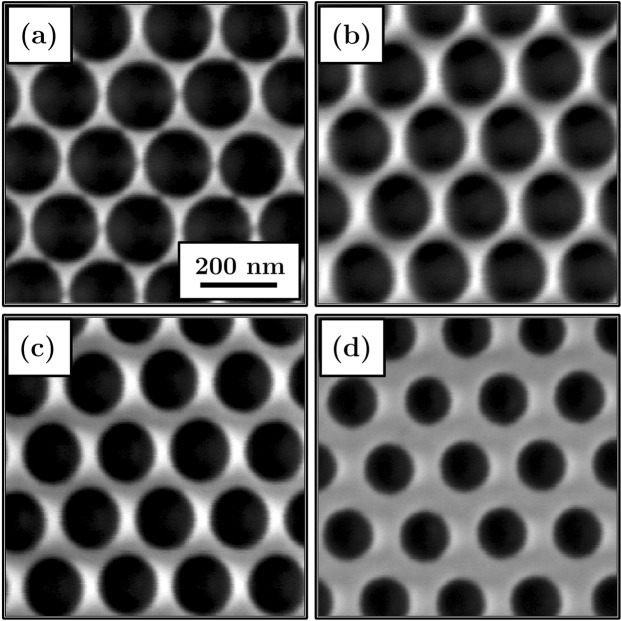
SEM images of the antidot arrays with a period of 202 nm. The measured mean antidot diameter is approximately (**a**) 182 nm, (**b**) 167 nm, (**c**) 157 nm, and (**d**) 128 nm.

**Figure 3:**
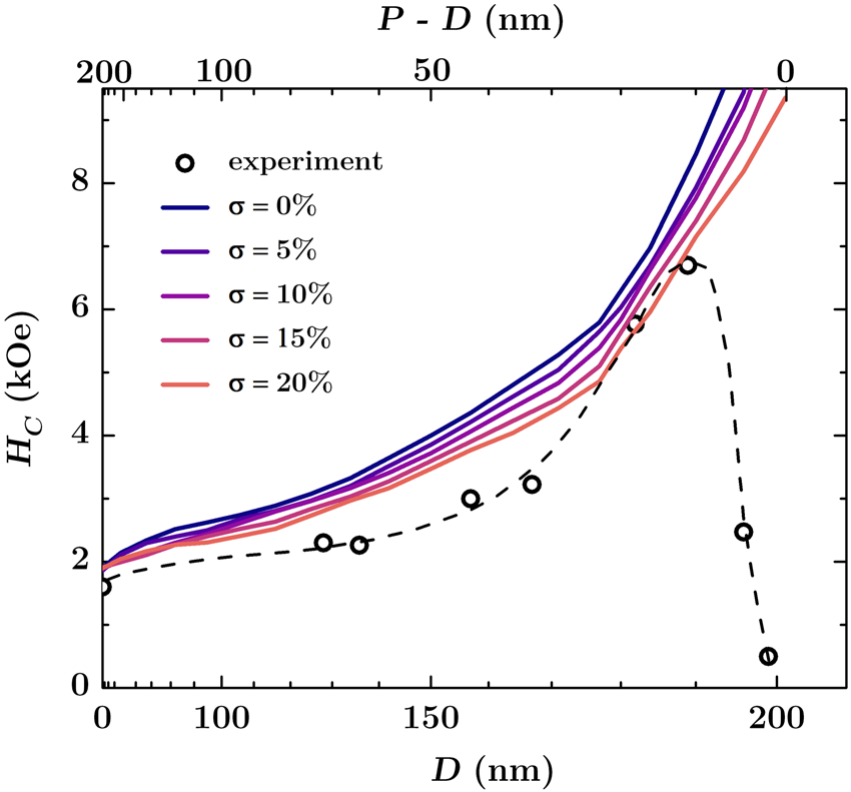
Coercivity for the out-of-plane direction of the magnetic field as a function of an antidot diameter (array with period of *P* = 202 nm). The continuous lines represent simulation results for a model with non-defected edges and different dispersion of antidot sizes expressed as a percentage of the diameter. The circles are the experimental results with dashed line as a guide to the eye. The scale over the top axis expresses distance between antidots calculated as the diameter subtracted from the period.

This micromagnetic model for a flat reference system with intrinsic defects was then used to analyze the influence of patterning on the magnetic properties of hexagonally ordered antidot arrays. The arrays was achieved in Co/Pd multilayers with total thickness of 12 nm, as detailed in the Experimental and Calculations section. Briefly, the highly ordered hexagonal lithography masks were prepared on silicon using self-assembling of polystyrene spheres. Afterward, the masks were plasma etched and covered by Co/Pd multilayers. After deposition, the spheres were removed from the samples, leaving behind the multilayer with a hexagonal array of circular holes (antidot array), as shown in Fig. 2. In the initial simulations the edges of the circular antidots were treated as ideal without any defects. For such system the domain wall pinning is expected, which should raise the coercive field values. Indeed, as seen in Fig. 3, the calculated out-of-plane coercivity for a patterned multilayer is significantly higher compared to the flat reference sample and *H*_*C*_ values strongly depend on the antidot diameter. A steady increase in the coercive field as the antidots diameter *D* increases is caused by the pinning effect, which prevents the free movement of domain walls through the material. The magnetization reversal is no longer governed by the long range movement of domain walls, but can be also carried out by magnetization rotation^28^. In this case, the nucleation field is lower than the depinning field of domain walls, which results in a drastic increase in the coercivity up to values 5 times bigger than for a non-patterned reference multilayer.

Similar changes are observed for the model including heterogeneity of antidot size. Results of this approach are presented in Fig. 3, where antidots diameters were varied according to a Gaussian distribution with standard deviation in the range 0–20% of their mean diameter. Such deviation is expected for most of the nanopatterning techniques. In particular, in the case of nanosphere lithography, the specification of nanoparticles suspension provided by the manufacturer claims the size variability of about 5%. As seen in Fig. 3, the random deviation of the antidot diameters from the nominal ones does not change the character of magnetization reversal, but reduces the resulting coercivity field up to 20% for the largest mean antidote size. This behavior is related to the accidental formation of wider necks (bridges) between neighboring antidots, which together create paths for easier propagation of the domain walls.

However, the SQUID magnetometry measurements carried out on Co/Pd antidot arrays indicate different behavior, showing that the growing trend of the coercivity breaks down when the distance between the antidots reaches values of approx. 20 nm, as shown by experimental results plotted in Fig. 3. It is seen that *H*_*C*_ reaches maximum and drops drastically to the values lower than those for the reference flat sample. Similar behavior was observed for the arrays with in-plane magnetization^29^, and it is generally explained by the presence of defects at the edges of the antidots. Usually the edge defects are considered to be a rim with a width of approximately 5 nm–30 nm having strongly reduced (even down to zero) anisotropy constant^19,30^. It has been shown that even with a rim of a few nanometers with decreased anisotropy, the nanostructures can exhibit edge nucleated reversal, which leads to a significantly lower switching fields^19^.

**Figure 4:**
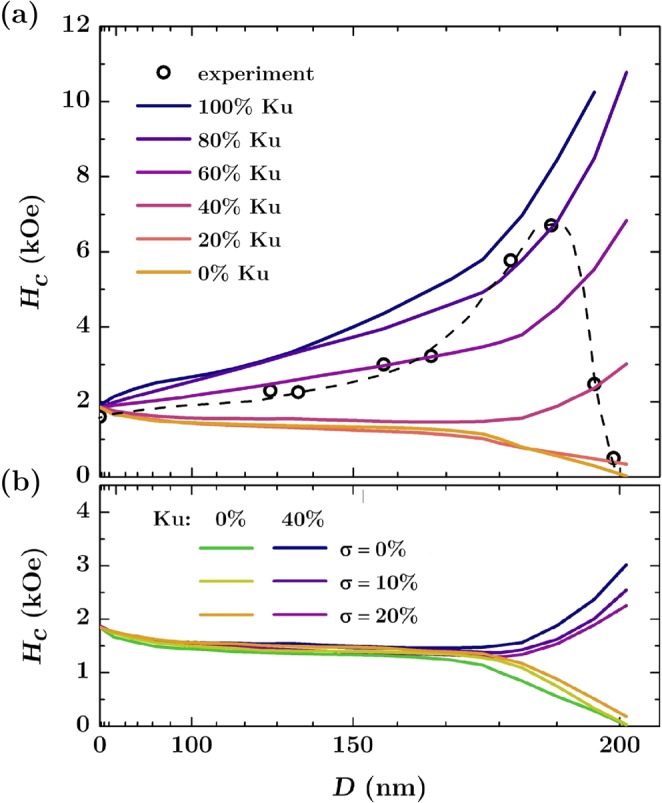
(**a**) Coercivity for the out-of-plane direction of the magnetic field as a function of an antidot diameter (array with period of 202 nm). The continuous lines represent simulation results for a model with defected edges and different anisotropy constants. The circles are the experimental results with dashed line as a guide to the eye; (**b**) Calculated *H*_*C*_ values for the model with defected edges having anisotropy constant reduced to 40% and 0% of the nominal value. Additional dispersion of the antidot diameters was included with different values of the standard deviation.

To check whether including this factor allows one to reproduce the experimental results for the arrays with perpendicular magnetization, we built a micromagnetic model of antidots with the rim having anisotropy constant reduced in the range of 0–100% of the nominal *K*_*U*_ for a material without defects. A width of the rim was assumed to be 15% of an antidot diameter. The obtained dependences of the coercive field are shown in Fig. 4a. As expected, we observe much smaller *H*_*C*_ values in comparison with a model that does not include the edge defects. Such behavior is a result of competition between the domain wall pinning appearing on the narrow necks (bridges) between the antidots, and the nucleation occurring at defected edges of the nanostructures. For the anisotropy constant of the rim smaller than 50% of the nominal value, the edges begin to dominate the reversal properties and facilitate fast domain nucleation, which leads to a significant reduction of the switching field. Although the obtained *H*_*C*_ values are closer to those measured in the experiment, no coercivity maximum is reproduced in this approach. A clear drop of *H*_*C*_ appears only when the anisotropy constant at the edges is reduced below 20%; however, *H*_*C*_ values are still far from those measured experimentally.

As shown in Fig. 4b, including heterogeneity of antidot sizes leads to similar results and only slightly changes *H*_*C*_ values. It allows us to state that a simple model with a uniform anisotropy reduction at antidot rims does not provide satisfactory quantitative results, and is not able to reproduce the magnetic reversal behavior of the system with PMA.

For this reason, we developed a model assuming that the defected material on the antidot edges shows not only reduced anisotropy constant but is also characterized by a reduced saturation magnetization. Such a change of net magnetization may be caused by an exposure of the material to atmosphere conditions and the diffusion of oxygen into the layer through unprotected edges. This, in turn, promotes the formation of various cobalt oxides. For example, CoO crystallizing in the rock salt structure is antiferromagnetic, similarly to Co_3_O_4_, which has a normal spinel structure with antiferromagnetic exchange between ions^31–33^. Both oxides have zero net magnetization due to the complete compensation of magnetic moments located on Co atoms. It can be thus expected that the partially oxidized edges of the antidots should behave like a weak ferromagnet with a significantly reduced *M*_*S*_ in comparison to the unaffected Co/Pd multilayer.

**Figure 5:**
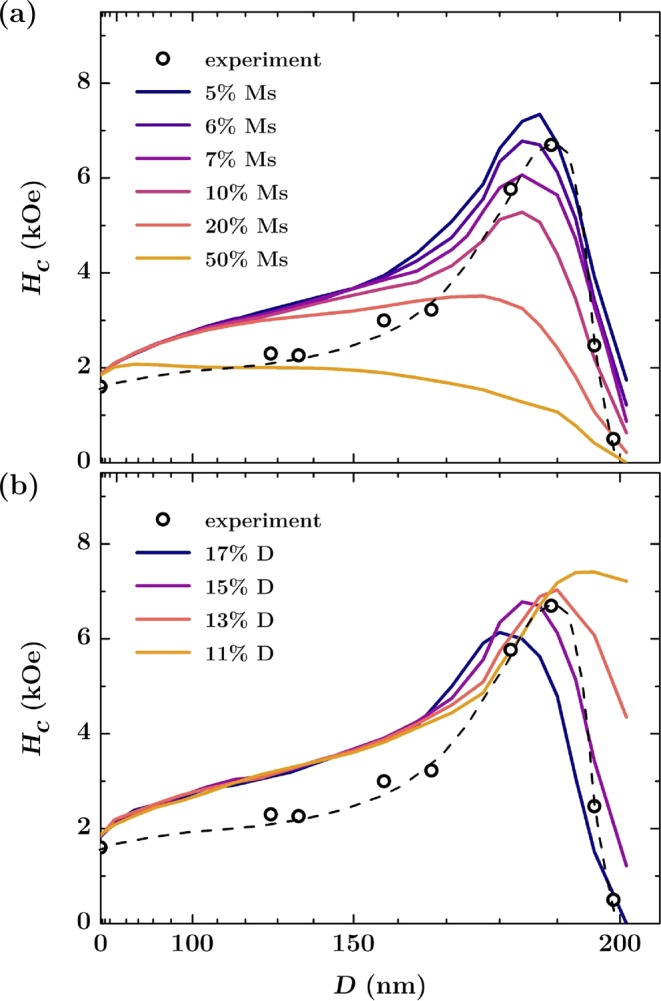
(**a**) Coercivity for the out-of-plane direction of the magnetic field as a function of an antidot diameter (array with period of 202 nm). The continuous lines represent simulation results for a model with defected edges with *K*_*U*_ = 0 and different magnetization saturations ranging from 5% to 50% of the nominal value for non-defected material. The distribution of the antidot size diameters was included in the model with the standard deviation of 5%. The circles are the experimental results with dashed line as a guide to the eye; (**b**) Simulated coercivity for a model with edges having *K*_*U*_ = 0, *M*_*S*_ = 6% of the nominal value for non-defected material and a different rim width ranging from 11% to 17% of the antidot diameter.

The dependences of the coercive field derived from this model are shown in Fig. 5a for different reduced *M*_*S*_ values assigned to the edges. The results correctly reproduce the measurements, and for *M*_*S*_ equal to or lower than 20% of the nominal value for material without defects, the coercive field dependence shows a characteristic maximum appearing for the antidot diameters in the range of 180–190 nm. Its amplitude is the higher, the smaller is the effective magnetic moment assigned to the edges. The best quantitative agreement with the experiment was obtained for *M*_*S*_ corresponding to 6% of the nominal magnetization value. In this case the adopted model predicted coercivity peak at 6.8 kOe, while the measured value was 6.7 kOe.

For the parameters of the antidot edges showing the best agreement with experiment (*K*_*U*_ = 0 J/m^3^, and *M*_*S*_ = 0.48 · 10^5^ A/m, which corresponds to 6% of the value for non-defected material), the coercive field dependences for variable rim thickness in the range from 11 to 17% of the antidot diameter was calculated, as shown in Fig. 5b. It is seen that the thickness of the defected edge strongly affects the changes of the coercive field, determining the height of *H*_*C*_ peak and its position. Domain nucleation at edges is easier for a thicker rim, resulting in a faster magnetization reversal associated with a more pronounced drop in the coercivity. Additionally, the simulations show that for the small size of the antidots it does not matter what rim thickness and edge magnetization we apply. For the diameter *D* in the range of 0–150 nm the *H*_*C*_ values are similar for each set of parameters as presented in Fig. 5, except the model with saturation magnetization reduced to 50% of the nominal value of *M*_*S*_ for flat films. This allows us to conclude that for arrays with small antidots, the presence of edge defects has no effect on *H*_*C*_ values and that the structural perturbations induced by the defects are too weak to change the magnetization reversal character of the entire system.

**Figure 6:**
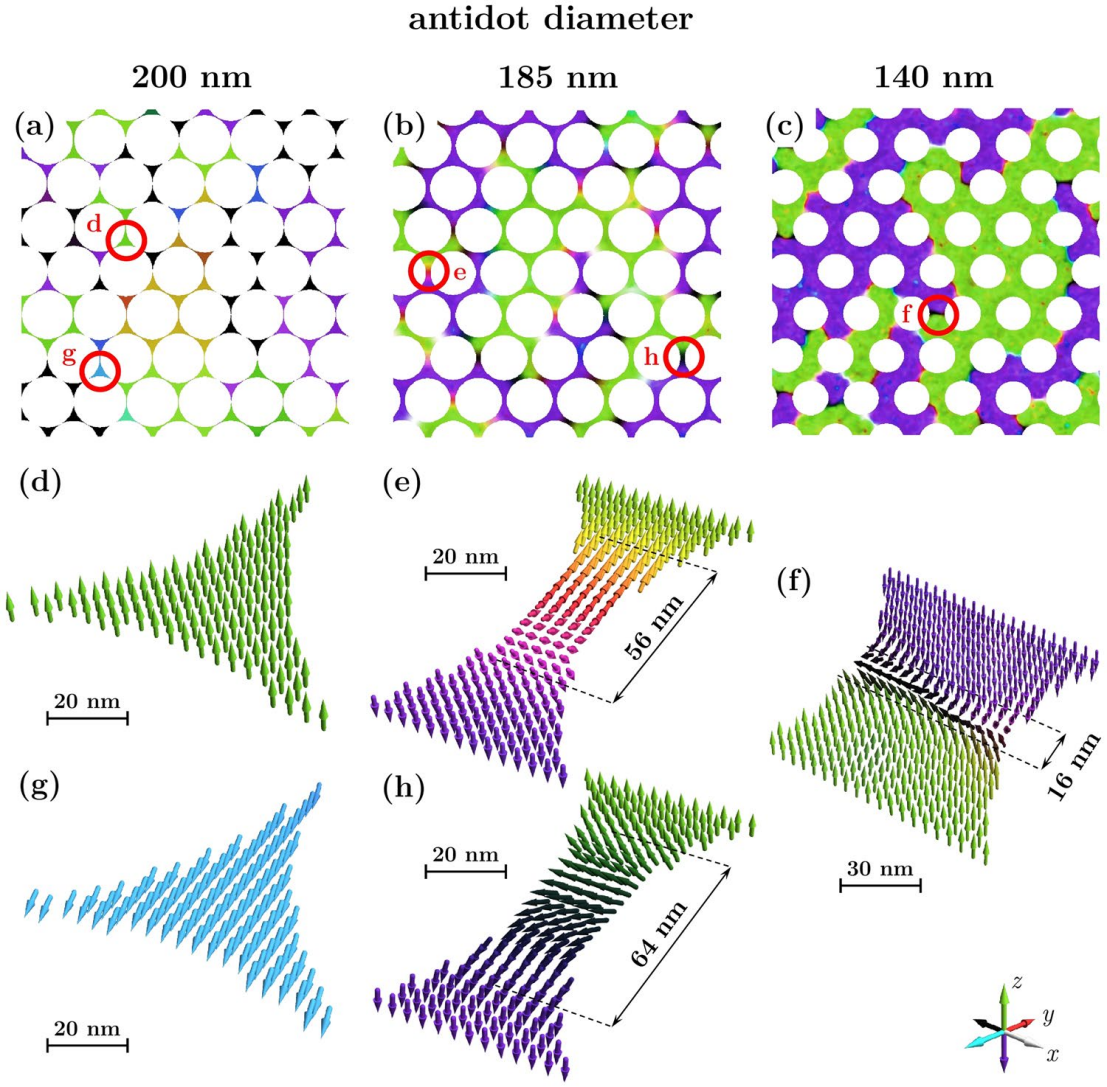
Simulated domain structure after ac demagnetization for the arrays of antidots with diameters (**a**) 200 nm, (**b**) 185 nm and (**c**) 140 nm. Pictures (**d**–**i**) show details of the structure for the regions marked with red circles.

**Figure 7:**
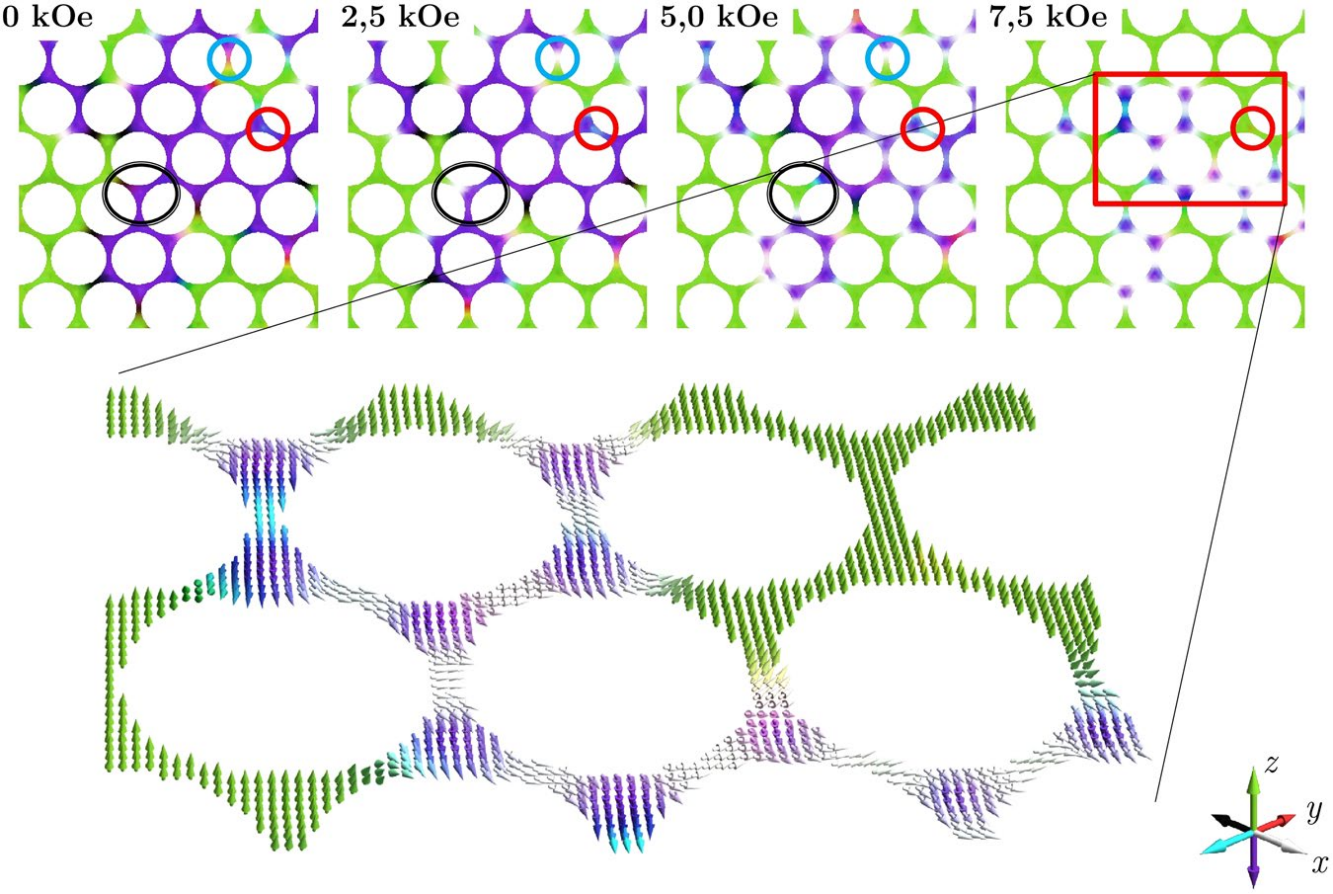
Evolution of the domain pattern for an array with an antidot diameter of 185 nm. Magnetic moment configuration was visualized for 4 different values of the external magnetic field. The red circle indicates the place where intrinsic defect is located, which is the source of edge nucleated reversal. The black circle surrounds the area where depinning and repinning processes for the domain wall are observed, while the blue circle indicates the place where transition from the Néel to the Bloch domain wall occurs. The region marked with a red rectangle at the last map is depicted separately below showing details of a bubble-like structure.

In order to better understand the magnetic reversal of the antidot arrays with the edge defects, we performed domain structure simulations for a model that best reproduces the measured changes of coercivity. The maps obtained in the zero external field after ac demagnetization are presented in Fig. 6 for three antidot sizes: 200 nm, 185 nm and 140 nm, together with details depicting the local configuration of magnetic moments. In addition, we examined the evolution of the domain patterns when applying an external magnetic field. The selected maps for arrays with an antidot diameter of 185 nm are shown in Fig. 7, while the entire sequences from the demagnetized state to the saturation are presented for all the cases as movies included in the Supporting Information.

The first domain pattern shown in Fig. 6a corresponds to an array with the big antidots almost being in contact with each other, with a low coercive field of 1,1 kOe. Since the width of the necks between the antidots is only 2 nm, the triangular regions between the three neighboring holes are weakly coupled and behave like isolated magnetic nanodots with monodomain structure. Only 60% of them retain the perpendicular configuration of magnetic moments (an example is presented in Fig. 6d), while for the remaining nanodots magnetic moments are parallel to the sample plane or tilted as shown in Fig. 6g. The trend to arrange magnetization within the sample plane is caused by the presence of edge and intrinsic defects, which strongly reduce the anisotropy of the entire nanodot and promote the in-plane configuration. As shown in Movie 2, during the reversal these islands quickly arrange magnetic moments along the external field, while the remaining “hard-switching” nanodots reverse through a coherent rotation supported by a rare nucleation of domains at triangle vertices.

**Figure 8:**
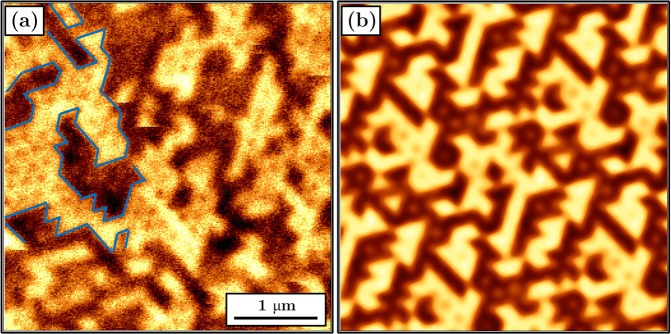
(**a**) MFM image for an array with an antidot diameter 182 nm taken in zero field after ac demagnetization. Selected domain walls were marked with a blue line. (**b**) Simulated MFM image for an antidot diameter of 185 nm corresponding to the magnetic moment configuration depicted in Fig. 6b. The MFM tip distance from the sample surface was 180 nm.

The pattern presented in Fig. 6b corresponds to array with the highest coercivity of 6,8 kOe. Simulated magnetic domain map in the demagnetized state shows that all the domain walls are pinned at necks between antidots. This behavior was confirmed by the MFM measurements presented in Fig. 8a, where the domain shape corresponds to the hexagonal structure of the array indicating that the antidot arrangement determines the location of domain walls. A similar picture was reproduced by a MFM simulation shown in Fig. 8b, calculated for the magnetic moment configuration depicted in Fig. 6b.

As shown in Fig. 6e, the domain walls are predominantly of the Néel type, in contrast to the Bloch walls observed in flat samples. Only approximately 10% of cases are intermediate Bloch-Néel walls, with an example depicted in Fig. 6h. Such a transition from the Bloch to the Néel walls in the nanopatterned systems has been already reported for thin nanowires with PMA, and has been explained by changes in energy relations^34–36^. As the width of the nanowire is decreased, the Néel domain wall becomes energetically favorable and a switch of wall type occurs below a width of 50–60 nm for Co/Ni and Co/Pd multilayers^34–36^. So far, such transition has not been yet observed in antidot arrays, but our simulations indicate that a neck between two antidots can behave like a short nanowire with a width determined by the array period and the antidot diameter. In the studied case its mean width is 15 nm, well below Bloch to Néel transition, which explains the observed character of the domain walls. One can also notice a significant increase in their width, which is approx. 60 nm, over 4 times more in comparison to a reference flat sample. This expansion is caused by the demagnetization fields and was explained by DeJong *et al*.^34^.

After application of the external magnetic field, the domain walls move by depinning and repinning from one neck to another, as shown in Movie 3. However, with the increasing field their type changes. The Néel walls disappear and transform into the Bloch walls whose energy is lower in the perpendicular field. As shown in Fig. 7, due to the high energy needed for domain wall depinning, the magnetization reversal is largely governed by the domain nucleation occurring at the defected edges of antidots. For fields higher than 6 kOe, the residual domain with an unfavorable direction of magnetic moments is seen. These do not consist of parallel magnetic moments, but instead form a network of magnetic bubbles presented in detail in Fig. 8. Similar bubble-like structures were reported by Marchenko *et al*. for the cobalt antidot arrays with in-plane magnetization^37^, but so far such configurations have not been observed for the systems with PMA.

The domain walls change their character for the smaller antidots, as presented in Fig. 7c for the antidot diameter of 140 nm. The walls are still pinned in the necks, but are bent and waved due to a bigger distance between the antidots which is 62 nm in this case. As shown in Fig. 6f, all of them appear as the Bloch walls with the width comparable to those for a flat reference multilayer, which agree with previous predictions^34–36^.

## Conclusions

In summary, we built a micromagnetic model of antidot arrays with perpendicular magnetic anisotropy which takes into account the polycrystallinity of the material, the intrinsic defects modeled as the “easy switchers” grains with reduced anisotropy, the extrinsic defect introduced by patterning, and the heterogeneity of the antidot sizes. By a careful, accurate selection of the grain size distribution, material parameters, and the dispersion of the easy-axis directions we successfully reproduced the magnetic features and the domain pattern for the reference flat multilayers. Next, we studied the magnetization reversal in the hexagonally ordered antidot arrays, and showed that the defects occurring at the rims of the nanostructures are necessary to reproduce the coercive field changes observed in the experiment. The simple model with a uniform anisotropy reduction at antidot rims, however, did not provide satisfactory quantitative results, and agreement with the experiment was achieved only after including the edge oxidation causing a local reduction of *M*_*S*_. The proposed model can be further developed, for example by taking into account the gradient of oxidation damages at the antidot edges or considering thermal fluctuations of magnetization. Davydenko *et al*. have also shown that in epitaxially deposited Co/Pd multilayers Dzialoszynski-Moriya interaction can additionally influence the magnetic reversal^38^.

Using the model we analyzed the magnetic domain pattern and found a transition from the Bloch to the Néel domain walls occurring for the arrays with the narrow necks between the antidots. The Néel walls dominated in demagnetized state; however, they disappeared during the application of the external magnetic field. Additionally, we predicted that the antidot lattice geometry connected with defected edges leads to the formation of structures, such as a network of magnetic bubbles, which are unstable in non-patterned flat films. These findings show that magnetic properties and domain configuration in nanopatterned systems are strongly determined by the defects, the heterogeneity of the nanostructure sizes and edge corrugations, and that such imperfections play a key role in the processes of magnetic reversal.

## Methods

### Preparation of the samples

Arrays of antidots were prepared on Si substrates using nanosphere lithography^39^. In the first step, polystyrene nanospheres with average diameters of 202 nm ± 10 nm were applied to the surface of water where a highly ordered hexagonal close packed monolayer of the spheres was created by self-assembly. Water was removed by slow evaporation leading to the deposition of the nanospheres on the Si substrate. Next, RF-plasma etching was used, resulting in a decrease in the sphere size, but maintaining their original positions and arrangement^40^. The plasma process was performed in oxygen and argon atmosphere at pressure between 0.1 and 0.2 mbar and temperature of approximately 30 °C with a chamber base pressure of 0.06 mbar. The final sphere size was determined by the plasma etching time and was chosen to be in the range of 60–100% of the initial sphere diameter. Structures obtained this way were characterized by the same period of 202 nm but variable distances between the polystyrene spheres ranging from 0 to 80 nm.

Arrays were further used as lithography mask during Co/Pd multilayer deposition. Pd (5 nm)/[Co (0.3 nm)/Pd (0.9 nm)]_10_ multilayers were deposited on the masks by sequential thermal evaporation at room temperature. A 2-nm capping layer of palladium was deposited on top of the films to prevent their oxidation. The working pressure in the preparation chamber was in the range of 10^−9^ mbar and the film thickness was controlled *in situ* by a quartz microbalance and *ex situ* by x-ray reflectometry. After deposition, the spheres were removed from the sample by ultrasonic assisted lift-off in toluene, leaving behind the multilayer with a hexagonal array of circular holes (antidot array). In the case of non-etched spheres, the antidots were touching each other and triangle dot arrays were obtained. Further details concerning mask and Co/Pd multilayers preparation are described in ref.^28^.

### Characterization

Magnetic measurements were performed using a superconductive quantum interference device (SQUID) magnetometer with a maximal magnetic field of ±70 kOe. All measurements were carried out in out-of-plane and in-plane geometry of the applied magnetic field. Magnetic imaging was performed using magnetic force microscopy (MFM), utilizing MFMR cantilevers (NanoWorld AG) covered with 40 nm thick hard magnetic cobalt alloy. Prior to the measurements the tip was magnetized along its vertical axis in the field of 5 kOe. The imaging was carried out at room temperature.

### Micromagnetic simulations

In order to model Co/Pd antidot arrays the MuMax3 software for micromagnetic simulations was exploited^41^. The simulations were performed at zero Kelvin neglecting thermal fluctuations with the effective material parameters homogeneous for the whole sample volume. Saturation magnetization *M*_*S*_ and intrinsic uniaxial anisotropy constant *K*_*U*_ were chosen according to magnetometry measurements for flat reference sample as 8.0 · 10^5^ A/m, and 9.0 · 10^5^ J/m^3^, respectively. The assumed exchange stiffness *A* was 10^−11^ J/m, based on previous reports^21,22,42^. Similarly, Landau-Lifshitz damping constant *α* = 0.15 was adopted according to the published results on Co/Pd multilayers with variable composition^43^. The simulations were performed for the samples with the size of 2048 nm × 2048 nm × 12 nm, discretized with the unit cell of 2 nm × 2 nm × 2 nm. The side length of the cell (voxel) was chosen to be smaller than the exchange length of 5 nm for Co/Pd estimated using the formula^44^:

2 $${l}_{ex}=\sqrt{\frac{2A}{{\mu }_{0}{M}_{s}^{2}}}.$$

In order to eliminate size effects, periodic boundary conditions were applied in the *x-* and *y-*directions.

**Figure 9:**
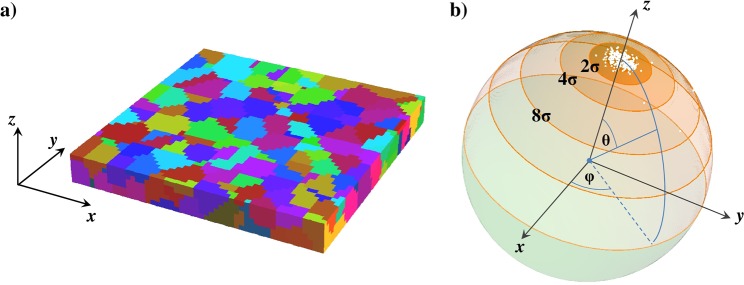
(**a**) Example of a division of a flat reference sample into grains obtained by the random number generator. Size of the region is 100 nm × 100 nm × 12 nm and each colour represents grain with a different orientation of the magnetization easy axis. (**b**) Distribution of the magnetization easy axes directions for grains showed in figure a) depicted in a spherical coordinate system. The *z* axis indicates a direction perpendicular to the surface of the sample, while the polar angle θ describes a deviation of the magnetization easy axis from the perpendicular direction. Each white dot on the graph represents an easy axis orientation for one grain. The regions designated as 2σ and 4σ represent the range of theta angles for which the probability of finding an axis orientation within them is 99.5% and 99.99%, respectively.

To mimic polycrystallinity and to consider intrinsic defects, the sample was divided into grains with the average size of 10 nm and standard deviation of 2.5 nm using 3D random Voronoi tessellation. The value of grain size was chosen according to the XRD and TEM measurements carried out on Co/Pd multilayers and reported before^45,46^. An exemplary division into grains for a selected region of a flat reference sample is shown in Fig. 9a. It was assumed that 10% of randomly selected grains is defected with the zero anisotropy constant *K*_*U*_. The presence of such grains could be caused by the lattice misalignment, high local roughness of Co/Pd interfaces, or the lack of continuity of the constituent layers, which is typical for thin multilayers deposited non-epitaxially on the rough substrates^47,48^. We assigned nominal value of the uniaxial anisotropy constant to the remaining 90% of the grains with a unique direction of *K*_*U*_ axis for each grain, which is expected for multilayers with distorted interfaces caused by heterogeneous growth. The orientation of the magnetization easy axes had a Gaussian distribution around the out-of-plane direction *θ* = 0 deg, with a standard deviation of *θ* = 5 deg (see Fig. 9b). Additional random distribution of easy axis directions was introduced for 2% of grains, according to the previous studies^20^, in order to include some irregularity of the real samples. In all simulations the external field was applied either in perpendicular or in parallel to the film plane with a tilting angle of *θ* = 3 deg.

Domain patterns in a demagnetized state presented in this paper were obtained using the following algorithm of relaxation. In the first stage, the system was magnetized out-of-plane by a high magnetic field of 10 kOe. Then, the field value was incrementally reduced with steps of *ΔH* = 100 Oe and switched to negative value amounting to 90% of the initial field strength. Next, the magnetic configuration was again relaxed. This procedure was repeated with a maximum field in each repetition being of 90% of the previous value until the external field strength was reduced to *H* = 500 Oe. Finally, the field was reduced to zero.

## Supplementary information

Video 1

Video 2

Video 3

Video 4

Supplementary Information

## Data Availability

The datasets generated and analysed during the current study are available from the corresponding author on reasonable request.
