# Supplementary material for: Magnetic reversal in perpendicularly magnetized antidot arrays with intrinsic and extrinsic defects
Source: Sci Rep. 2019 Sep 16;9:13276. doi: 10.1038/s41598-019-49869-5 (PMC6746764; doi:10.1038/s41598-019-49869-5)
Supplement: Supplementary file 5 — Supplementary Information [file 41598_2019_49869_MOESM5_ESM.pdf]

Supplementary Materials

Magnetic reversal in perpendicularly magnetized antidot arrays with intrinsic and extrinsic defects

Michal Krupinski*, Pawel Sobieszczyk, Piotr Zieliński, Marta Marszałek

Institute of Nuclear Physics Polish Academy of Sciences, Radzikowskiego 152,

31–342 Kraków, Poland

List of videos showing magnetic reversal of flat Co/Pd multilayers and antidot arrays with period 202 nm. In all cases external magnetic field was applied in the direction perpendicular to the sample plane and changed with step of 25 Oe:

- Video 1. Flat reference sample. The range of external field was 0 – 2.5 kOe.
- Video 2. Array with antidot diameters of 200 nm. The range of external field was 0 – 3.2 kOe.
- Video 3. Array with antidot diameters of 185 nm. The range of external field was 0 – 9.0 kOe.
- Video 4. Array with antidot diameters of 140 nm. The range of external field was 0 – 2.5 kOe.

Changes of magnetization caused by patterning

Changes of M_S value in antidot arrays are associated with two factors. The first is the reduction of the area covered with magnetic material as the diameter of antidot increases. The larger the antidots, the smaller the contribution of the sample surface coated with Co/Pd multilayer. For a hexagonal antidot arrangement, this coverage ratio can be estimated using the following relation:

$$c_1 = 1 - \frac{\pi D^2}{2\sqrt{3}P^2}$$

where D is the antidot diameter, and P is a period of the array (in our studies P = 202 nm). The smallest value of c_1 corresponding to the strongest reduction of M_S can be obtained for the biggest antidot sizes, which in our case are 200 nm. For these parameters, the resulting M_S is 11% of the nominal M_S value for non-patterned film.

The second factor responsible for the reduction of M_S is the oxidation of the rim of the antidots. Because the rim has magnetization close to zero, it reduces the average magnetization of the entire layer. This can be expressed as:

$$c_2 = \frac{P^2\sqrt{3} - 2\pi\left(\frac{1}{2}D + R\right)^2 + 2\rho\pi R(D + R)}{P^2\sqrt{3} - \frac{1}{2}\pi D^2}$$

where D is the antidot diameter, P is a period of the array (in our studies P = 202 nm), R is width of the rim, and ρ is a percentage of the nominal Ms value associated with the rim. The expression is valid only when $D + 2R < P$ but allows to estimate the reduction of the Ms value for most of the analyzed range of antidot sizes. For example, when $\rho = 0.06$, D = 160 nm and R = 20 nm (exemplary values taken from Fig. 5), we get $c_2 = 0.3$.

Thus, the resultant M_S value for the entire antidot array can be estimated as:

$$M_S = c_1 c_2 M_{S,nom}$$

where Ms,nom is nominal Ms value for non-patterned film and was adopted according to magnetometry measurements for flat reference sample as $8.0 \cdot 10^5$ A/m.

Magnetization components

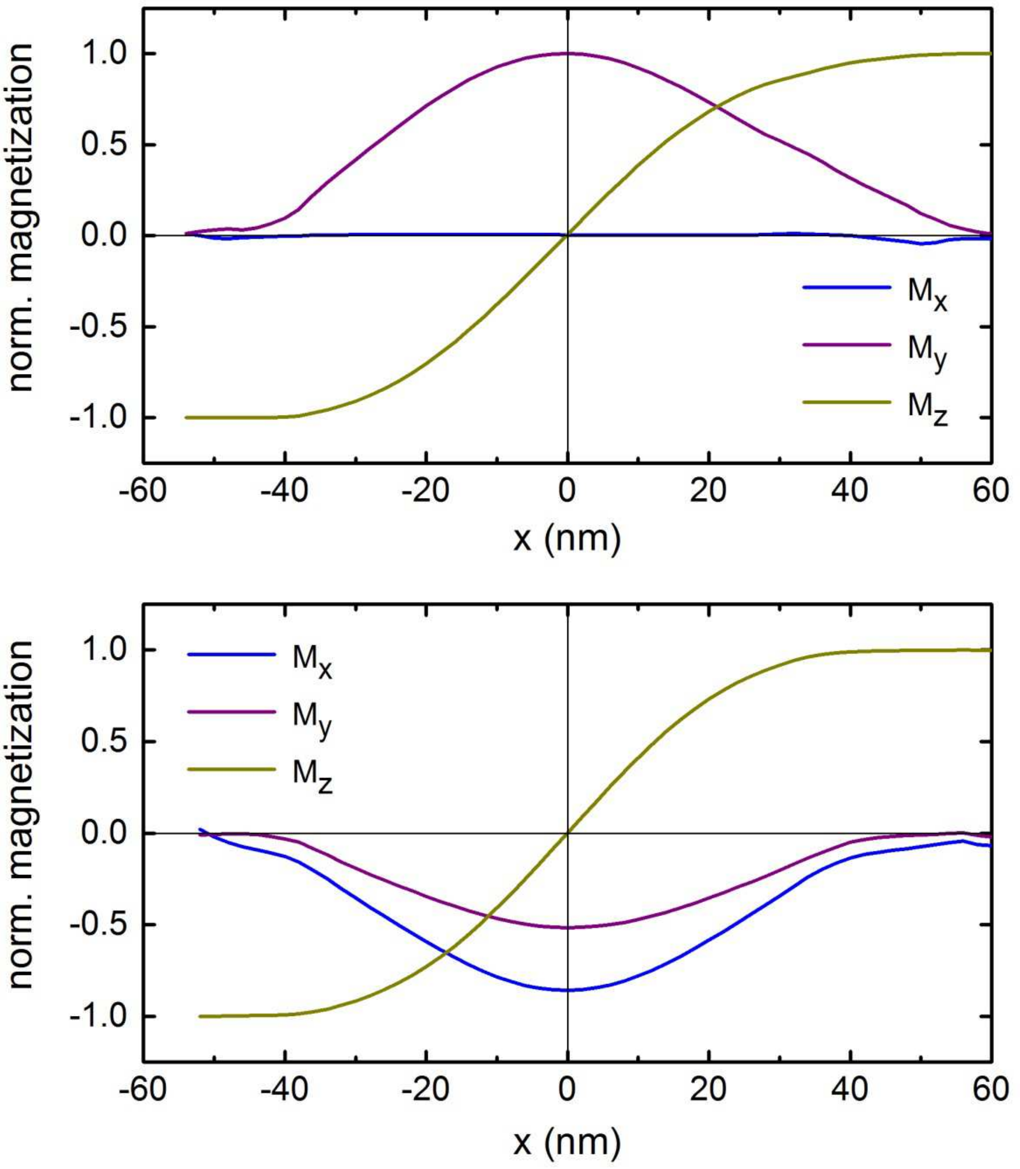

Figure S1. Magnetization components as a function of distance *z* along the neck between two antidots for (a) the Neel wall presented in Fig. 7e, and (b) for intermediate Bloch-Néel wall depicted in Fig. 7h.
